# Biodiversity and emerging infectious threats: the microbial dark matter of Southwest China

**DOI:** 10.3389/fmicb.2026.1846062

**Published:** 2026-06-02

**Authors:** Nan Guo, Zhiguo Liu, Zhenjun Li, Jianguo Xu

**Affiliations:** 1 Research Center for Reverse Microbial Etiology, Workstation of Academician, Shanxi Medical University, Taiyuan, China; 2 School of Public Health, Shanxi Medical University, Taiyuan, Shanxi, China; 3 State Key Laboratory of Infectious Disease Prevention and Control, National Institute for Communicable Disease Control and Prevention, Chinese Center for Disease Control and Prevention, Beijing, China

**Keywords:** cross-species transmission, microbial dark matter, novel pathogens, Southwest China, zoonotic diseases

## Abstract

Southwest China is a global biodiversity hotspot, and its complex and diverse ecosystems harbor vast amounts of “microbial dark matter.” This paper systematically examines the distribution characteristics of microbial dark matter in hosts such as arthropods, mammals, and birds, as well as in environments including soil, hot springs, and high-altitude lakes, with a particular focus on the cross-species transmissibility and pathogenic potential of emerging pathogens. Research indicates that new microbial species in the Southwest exhibit significant geographic concentration and host specificity: Yunnan Province is a core hotspot, while the Tibet Autonomous Region contributes a wealth of microbial resources due to its extreme environments, with arthropods and mammals accounting for the highest proportion of novel species. Regarding public health risks, eight novel pathogens with evidence of human infection have been identified, spanning the three major groups of viruses, bacteria, and parasites. The cross-species transmission potential of some pathogens (such as DPRV rhabdovirus, PPV arenaviridae, Luxi hantavirus, Banna virus and a novel *Babesia* species) has been confirmed through serological surveys or molecular testing. Deepening the exploration of microbial dark matter and risk early warning in this region will provide critical scientific support for public health safety monitoring.

## 1 Introduction

Microorganisms are the most diverse and widely distributed form of life on Earth, capable of colonizing virtually every ecological niche. However, the bacteria we are currently able to culture account for less than 1% of the total, which means that human understanding of the microbial world remains extremely limited (Yarza et al., 2014). This vast, unexplored realm is known as “microbial dark matter” – a world of immense diversity that remains beyond human grasp and can only be glimpsed through non-culture-based techniques (Jiao et al., 2021). Microorganisms play a central role in regional material cycles and ecosystem stability, but they also serve as a major reservoir for novel pathogens. The global outbreak of COVID-19 demonstrates that unknown microorganisms pose a constant threat to humanity and, once they breach the species barrier, can trigger a global public health crisis in a short period of time.

Southwest China encompasses a variety of ecosystems, including the Qinghai-Tibet Plateau, the Hengduan Mountains, the Yunnan-Guizhou Plateau, and the Xishuangbanna tropical rainforest, and is recognized globally as one of the world’s biodiversity hotspots (Mi et al., 2021). With its complex terrain and diverse climate zones, this region has not only given rise to a unique array of flora and fauna, but also harbors a vast microbial community (Sessitsch et al., 2023). In recent years, the discovery of new microbial species in Southwest China has been frequent, creating an urgent need for a comprehensive, regional-level review to clarify research progress and future directions. Therefore, in this review, we will outline the current state of research on the microbial dark matter in Southwest China, focusing on both established findings and areas requiring further investigation. First, we will summarize research progress on unknown microorganisms in various hosts and environments across the Southwest; second, we will focus on elucidating the ecological distribution and pathogenic potential of key pathogens; and finally, we will explore the critical role of the ongoing exploration of the microbial dark matter in public health risk prevention and control.

## 2 Microbial diversity of host origin

### 2.1 Arthropods

Arthropods, particularly blood-feeding species, serve as key vectors for the transmission of numerous microorganisms due to their widespread geographic distribution, close contact with humans and other animals, and their biological characteristic of feeding on blood (Li C. et al., 2025; Zhu Y. et al., 2025). Globally, insect-borne viruses account for more than 17% of known human infectious diseases; major pathogens such as dengue, Zika, and West Nile viruses are all transmitted by these vectors, representing a sustained public health burden in tropical and subtropical regions (Jones et al., 2008; Weaver and Reisen, 2010). In southwestern China, the complex terrain and diverse climatic conditions provide a suitable habitat for vectors such as mosquitoes, ticks, and fleas, making them key carriers and vectors for the spread of new microbial species in the region.

#### 2.1.1 Mosquitoes

As important vectors of disease, mosquitoes harbor complex viral communities, including both mosquito-specific viruses and arboviruses capable of infecting humans. Research on the diversity of mosquito viruses has made significant progress both nationwide and in the southwestern region. Pan et al. (2024) conducted metatranscriptomics analysis of 2,438 mosquitoes representing 81 species nationwide, identified 393 mosquito-associated viruses (including 7 arboviruses), and revealed the distribution patterns of mosquito viral diversity in China. Focusing on the Southwest region, 52 viruses from 15 families (including 19 newly discovered viruses) have been identified in five species of mosquitoes in Yunnan Province (Li C. et al., 2023); a total of 162 viral fragments (including 22 unidentified viruses) were identified among 40,000 adult mosquitoes collected from eight ecological zones in Guizhou Province (Linghu et al., 2025). These studies confirm that mosquitoes are key vectors for the discovery of new viral species in the southwestern region, and the diversity of viruses they carry far exceeds previous understanding.

Identifying the functional characteristics of cultivable microorganisms in mosquitoes is equally important. For example, the newly identified species *Rosenbergiella* sp. YN46, isolated from the gut of *Aedes albopictus* in Yunnan, exhibits a higher natural prevalence in mosquito populations in areas with low dengue fever prevalence, suggesting that this strain may have the potential to block the transmission of mosquito-borne viruses, thereby offering new insights for biological control (Zhang et al., 2024). This highlights the protective role of newly discovered microbial communities, which can reduce the spread of pathogens through competitive exclusion.

#### 2.1.2 Ticks

As a major global vector of infectious diseases, ticks pose a threat second only to mosquitoes (Maldonado-Ruiz, 2024). Since the 1980s, a total of 33 novel tick-borne pathogens have been identified in mainland China, including *Rickettsia* species of the spotted fever group, *Anaplasma* species, the Borrelia complex, and *Babesia* species; 15 of these have been confirmed to cause human disease (Fang et al., 2015).

Tick-borne pathogens in the Southwest region exhibit regional characteristics: on the Qinghai-Tibet Plateau, 492 and 294 potentially novel species were identified in ticks and fleas parasitizing the Himalayan marmot, respectively, including various pathogenic agents such as phagocytosis-prone anaplasmas, novel ehrlichiae, bartonella, and rickettsiae (Dong et al., 2023). A study of ticks in the China-Myanmar border region has identified two novel species of *Babesia* (Li et al., 2020). Surveys conducted in Yunnan, Guizhou, and Guangxi have further expanded the regional pathogen spectrum, with a novel species of *Rickettsia* identified from tick samples collected from both wild and domestic animals (Wang et al., 2021). A novel species of the genus *Ehrlichia* has been discovered in *Boophilus microplus* found on livestock in Guizhou (Liu et al., 2025); two new viruses were detected in 1,286 ticks collected from wild animals and livestock in Guangxi (Chen P. et al., 2025). Together, these studies indicate that the southwestern region is home to a wide variety of tick-borne pathogens, underscoring the need for ongoing, region-specific surveillance.

#### 2.1.3 Other arthropods

In addition to mosquitoes and ticks, many other arthropods are also important carriers and vectors of emerging microorganisms. Regarding pathogen transmission, fleas, as obligate blood-feeding insects, carry a diverse range of viruses that cannot be overlooked–30 new RNA viruses have been identified in fleas found on the bodies of Himalayan marmots on the Qinghai-Tibet Plateau, and six viruses were found to be shared between ticks and fleas, suggesting the existence of cross-vector transmission (Zhu W. et al., 2025).

Midges, as widely distributed blood-feeding insects, are often confused with mosquitoes. A survey conducted across 15 regions in Yunnan revealed that midges and mosquitoes in the same areas share similar viral community structures. Not only were at least 10 major pathogenic viruses detected in midges, but a large number of novel viruses were also identified, including 21 segmented viruses of Flaviviridae, 180 viruses of Monjiviricetes and 130 viruses of Bunyavirales (Liu et al., 2021). Regarding the risk of cross-species transmission, the novel Oya virus isolated from midges in Yunnan has already shown a high seroprevalence rate in local pig populations (Meng et al., 2023).

With regard to parasitic arthropods, a total of 144 RNA viruses were identified in samples of bat flies, ticks, mites, fleas, and other arthropods collected from the bodies of 686 bats in Yunnan Province; of these, 138 were novel viruses, and the viral composition exhibited a highly specific distribution across different arthropod groups (Xu et al., 2022). Insect-pathogenic fungi occupy a unique position in the regulation of host behavior; the discovery of six novel species of “zombie ant fungi” in Yunnan is notable for their ability to manipulate the behavior of their host ants (Tang et al., 2023).

Overall, the diverse arthropod communities in Southwest China constitute a vast reservoir of pathogens; they serve as a key source for the discovery of new viral species and are a primary driver of cross-species transmission of vector-borne diseases. Their microbial diversity and overlapping ecological niches represent a potential risk to regional public health.

### 2.2 Mammals

Mammals, particularly small mammals such as rodents and bats, have become important reservoirs of emerging microorganisms due to their high species diversity, widespread distribution, and overlap with human activity areas. A study synthesizing data on small mammals and associated microbial infections in China from 1950 to 2021 indicates that these animals share extensive contact with numerous microorganisms with the potential to cause disease in humans, underscoring the importance of sustained surveillance (Chen J. et al., 2025). In recent years, several new viruses with unique evolutionary lineages have been discovered in various small mammals across 20 provinces nationwide. It has been confirmed that cross-species host jumps occur frequently in viral evolution, significantly expanding our understanding of viral diversity in China’s wildlife (Wu et al., 2018). Therefore, strengthening systematic monitoring and research on mammals–especially small mammals–is of critical importance for the early warning and prevention and control of the spread of zoonotic diseases.

#### 2.2.1 Rodents

There are currently more than 2,000 species of rodents, accounting for approximately 40% of all mammals; they are one of the most diverse and widely distributed groups of mammals (Huchon et al., 2002). As a key component of the ecosystem and a natural reservoir for zoonotic pathogens, mapping their pathogen diversity is of great significance for the early warning of emerging infectious diseases (Chen et al., 2017).

As key reservoirs of pathogen diversity, new discoveries regarding the microbial composition of rodents continue to underscore their unique role in zoonotic diseases. Regarding viruses, Hu et al. (2014) analyzed 968 anal swab samples from small mammals and found that astroviruses exhibit high genetic diversity among hosts. Numerous new viral lineages were detected in 10 bat species, and new viral groups were also identified in three wild rodent species, with *Eothenomys cachinus* harboring particularly high astrovirus diversity (Hu et al., 2014). With regard to bacteria, a novel *Ehrlichia* has been identified in rodents in Guizhou Province; phylogenetic analysis confirms that it represents a distinct evolutionary lineage (Lu et al., 2022); a novel species of *Escherichia marmotae* isolated from the feces of Himalayan marmots on the Qinghai-Tibet Plateau has been experimentally confirmed to possess the ability to invade cells, suggesting that it is a potential pathogen (Liu et al., 2019).

Rodents are the primary natural hosts of important pathogens such as hantavirus. Hantavirus cause hemorrhagic fever with renal syndrome in Eurasia and hantavirus pulmonary syndrome in the Americas (Chen et al., 2023). In the Fugong region of Yunnan Province, China, researchers observed a high prevalence of hantavirus infection among small mammals and identified a novel hantavirus–Fugong virus (FUGV)–in the small oriental vole (Ge et al., 2016b). Monitoring data from Yunnan show that the positivity rates for Hantavirus in bats and rodents were 27.49% and 1.25%, respectively, and a new lineage of Seoul virus (SEOV) was identified (Hou et al., 2024). Similarly, in the Yunnan region–an area with high levels of human disturbance–it has been established that *R. tanezumi* serves as a key reservoir and vector for various zoonotic viruses, including Porcine bocavirus, hantavirus, cardiovirus, and lyssavirus; furthermore, new viruses with the potential to infect humans have been identified (Kane et al., 2024). These findings not only reveal the hidden distribution and genetic diversity of Hantavirus in China, but also suggest the impact of human activities on the composition of the viral community and the risk of spillover.

*Yersinia pestis* is a globally distributed, naturally occurring pathogen that causes the plague. Genomic studies indicate that Yunnan Province in China is the origin of its Oriental subspecies, providing a basis for analyzing global transmission routes (Morelli et al., 2010). A previously undefined monophyletic clade, 1.IN5, was isolated from rodents in Heqing County, Yunnan, revealing a unique evolutionary lineage in the region (Shi et al., 2021). A novel *Yersinia* bacteriophage isolated from soil at a plague epicenter in Yunnan can inhibit the spread of plague by modulating microbial communities, offering a potential tool for ecological control (Long et al., 2025). These studies have established Yunnan’s pivotal role in the origin and evolution of *Yersinia pestis*.

Rodents hold immense potential for the discovery of novel species and serve as an indispensable component of surveillance networks for emerging pathogens; both the microbial resources they harbor and the potential risks they pose warrant close attention.

#### 2.2.2 Bats

Bats account for more than 20% of the world’s mammalian species, making them one of the most diverse groups and a natural reservoir for numerous viruses. Due to their unique immune characteristics, extensive species and geographic distribution, and relative phylogenetic proximity to humans, the risk of viruses carried by bats spilling over into human populations is significantly higher than that of other mammals (Demian et al., 2024). Bats also carry a large number of unknown pathogens, and several high-profile emerging infectious diseases have been linked to them; and this species is abundant and diverse, with a wide geographic distribution, and Yunnan Province in China is one of its primary habitats (Calisher et al., 2006; Han et al., 2015). A nationwide bat virology study has revealed remarkable viral diversity. The sequencing of 4,143 bat samples representing 40 species from 52 locations across China has expanded the known diversity of bat RNA viruses by more than 3.4 times, and found that bats inhabiting areas near human settlements carry a greater number of viruses capable of infecting humans and livestock (Yan et al., 2025).

Bats are important reservoir hosts for coronaviruses, and research on this topic has become a major focus in the field of emerging infectious diseases. On a national scale, Han et al. (2023) tested over 13,000 bat samples from 14 provinces and identified a total of 1,141 coronavirus strains, including 11 novel species, with the highest viral diversity observed in Yunnan. A systematic survey by Wu et al. (2023) further identified 146 novel sarbecovirus, and SARS-CoV-related coronavirus strains from the southwestern border region showed the highest sequence homology with human viruses, suggesting that the transmission network may extend to Southeast Asia. Focusing on the Yunnan region, research on bat coronaviruses has also yielded several breakthroughs: analysis of 61 bat samples has identified five new types of coronaviruses (Li et al., 2024); not only were multiple novel coronaviruses detected in six bat populations in abandoned mines in Mojiang County, but mixed infections were also observed, highlighting the potential risk of bats serving as a breeding ground for viral evolution (Ge et al., 2016a); in addition, 11 novel viral strains discovered among various bat species in a certain cave have been experimentally confirmed to be closely related to SARS-CoV, providing key evidence regarding the virus’s origin and evolution (Hu et al., 2017). Together, these studies indicate that Yunnan and the southwestern border regions constitute a hotspot for coronavirus diversity and represent a key frontier for monitoring the risk of future emerging coronaviruses.

In addition to coronaviruses, bats in the Yunnan region have been found to carry a variety of other high-risk pathogens. In kidney tissue samples from 142 bats representing 10 species within the province, researchers detected 20 novel viruses (including two new henipaviruses), a novel protozoan parasite, and a new bacterial species with high abundance (Kuang et al., 2025); among bats and their cutaneous parasites along the China-Myanmar border, the prevalence rates of *Bartonella* were 22.96% and 62.50%, respectively, and a novel species, “Candidatus *Bartonella dianxisis*” was identified (He et al., 2025); four new fungal species have been discovered in bat carcasses found inside caves; among them, the genus *Neocosmospora* is associated with fungal poisoning in humans and animals, and related infection cases are on the rise (Karunarathna et al., 2020). The aforementioned studies indicate that bats in the Yunnan region serve not only as a major reservoir for coronaviruses but also carry a variety of other pathogens that pose potential zoonotic risks, underscoring the region’s critical role in the surveillance and early warning of emerging pathogens.

#### 2.2.3 Other mammals

In addition to the aforementioned groups, many other mammals are also important subjects of research on microbial diversity. The Eulipotyphla, the third-largest group of mammals in terms of species richness, is one of the major potential sources of emerging human infectious diseases. A systematic analysis of public databases reveals that these animals harbor a total of 941 microbial species, 60% of which are viruses. Furthermore, human-associated viruses found in shrews and hedgehogs are phylogenetically very closely related to human viral strains, suggesting a risk of bidirectional transmission (Li H. et al., 2025). In southwestern China, studies of the Yunnan tree shrew and 13 other insectivorous species have further confirmed the extremely high viral diversity within this group; more than 60 potentially novel viruses have been identified, highlighting their potential for cross-species transmission (Yang et al., 2025; Zhou et al., 2024).

The Plateau Pika, a species endemic to the Qinghai-Tibet Plateau, also serves as a significant reservoir for pathogens. Systematic virological studies have not only revealed a vast viral reservoir containing over 290,000 viral units but have also identified 32 viruses capable of cross-species transmission, 22 of which pose a potential risk of spillover (Lu et al., 2025). The discovery of a novel arenavirus–Plateau pika virus, PPV–is particularly noteworthy, as this virus can replicate in mammalian cells and cause fatal encephalitis in immunodeficient mice; the presence of seropositive cases among local residents suggests that cross-species transmission may have occurred (Luo et al., 2023). In addition, Plateau Pikas are widespread carriers of alpha and beta coronaviruses (Xu et al., 2024), and serves as a natural intermediate host for the fine-grained echinococcus tapeworm (Chen W. et al., 2025).

As a key protected wildlife species, the pangolin’s viral diversity warrants close attention. Studies on smuggled pangolins and native pangolins in Guangxi have revealed the presence of not only several vertebrate-associated viruses reported for the first time, but also viruses capable of infecting humans and mosquito-borne zoonotic viruses. This suggests that illegal trade may increase the risk of cross-species pathogen transmission (Shi et al., 2022; Zhang D. et al., 2022).

Research on viral diversity in wildlife continues to push the boundaries of our understanding. A survey of 2,175 wild animals in southern China (including Tibet and Sichuan) identified mammalian viruses from 27 families, including a novel genus of Bornaviridae, a new clade of Embecovirus and a new genus of arenaviruses, and revealed potential cross-species transmission between wild animals and domestic livestock (Cui et al., 2023). Nine novel parvoviruses have been identified in the feces of herbivores on the Qinghai-Tibet Plateau (Chen Y. et al., 2025); a novel strain of Crimean-Congo hemorrhagic fever virus was identified in liver tissue from *C. milneedwardsii* that died in Yunna (Wang et al., 2024). This suggests that wild even-toed ungulates may play a role in the natural circulation of the virus, posing a potential risk of spillover.

Focusing on Yunnan, systematic research has further confirmed the region’s high-risk status. A total of 5,346 viral gene fragments were identified in 16 species of wild small mammals in Xishuangbanna, 11 of which are potential zoonotic pathogens (Guo et al., 2025); a total of 162 viruses have been identified in 38 mammal species in Yunnan, 102 of these are novel species, and 24 are associated with human pathogens (Feng et al., 2025). In addition, six novel species of rickettsia and one novel species of *Borrelia recurrentis* have been reported in the region (Du et al., 2024a,b).

The above studies indicate that mammals–particularly small mammals with high ecological niche overlap with humans–constitute a vast and dynamic reservoir of pathogens. Due to its complex ecological environment and dense animal-human contact interfaces, southwestern China has become a key region for the emergence and spillover of pathogens responsible for emerging infectious diseases.

### 2.3 Birds, reptiles, and aquatic animals

As key components of ecosystems, the microbiomes of birds, reptiles, and aquatic animals hold a wealth of untapped resources.

Birds, particularly long-distance migratory birds and widely distributed resident birds, play a unique role as bridges in the spatiotemporal transmission of microorganisms. As mobile hosts, birds connect different geographic regions and ecosystems, and the microbial communities they carry are closely linked to their migration patterns, feeding habits, and habitats. Not only were 50 undescribed species and 161 previously unknown lineages identified in the feces of vultures on the Qinghai-Tibet Plateau, but vultures were also found to be important reservoir hosts for *Clostridium perfringens* (Meng et al., 2017). Combined with their unique role in sky burial rituals, this suggests that there may be a distinct transmission chain. Research on the avian viral genome continues to expand our understanding: a novel Pegivirus has been identified in three species of passerine birds on the Qinghai-Tibet Plateau, marking the first time the host range of this virus has been extended from mammals to birds (Zhu et al., 2022); a novel hepatovirus has also been detected in migratory birds in the Southwest region (Luo et al., 2025). Poultry populations are also of significance for surveillance; for example, the discovery of a novel Pegivirus in a goose flock in Sichuan has further enriched the genetic diversity of this viral genus (Zhen et al., 2021).

Research on the microbiome of reptiles is still in its infancy, but unique and diverse viral communities have already been identified within their bodies. Due to their greater phylogenetic distance from mammals, reptiles may harbor highly specialized or as-yet-undiscovered viral lineages. Members of multiple viral families, including the Adenoviridae and Circoviridae, have been identified in oral and fecal samples from several snake species in Yibin, Sichuan Province. The iflavirus and foamy virus were first reported in *Protobothrops mucrosquamatus*, phylogenetic analysis suggests that these new viruses may represent distinct evolutionary lineages (Liu et al., 2023).

Aquatic animals, particularly fish inhabiting the unique aquatic environments of high-altitude regions, provide a crucial window into understanding microbial adaptation and evolution in extreme environments. A total of 28 potential novel viruses have been identified from various healthy fish species in the Lhasa River in Tibet; 22 of these may be associated with vertebrates and belong to seven viral families, suggesting that fish may serve as reservoirs for a large number of unknown viruses (Xi et al., 2023).

The aforementioned studies indicate that birds, reptiles, and aquatic animals play a crucial role in research on microbial diversity; the “dark matter” contained within their microbiomes not only expands our understanding of biological diversity but also offers new perspectives for assessing ecological safety and public health risks.

Overall, the research in this section reveals several cross-disciplinary patterns. From an ecological perspective, hotspots for the discovery of novel pathogens are concentrated at the interface between blood-feeding arthropods and mammals–particularly in areas where mosquitoes interact with primates, ticks with rodents and livestock, and bats with humans–as well as in transitional landscapes such as the China-Myanmar border and the margins of Yunnan and the Qinghai-Tibet Plateau. In these regions, high host diversity, frequent interspecies contact, and intensive human activity collectively facilitate pathogen maintenance and spillover. Taxonomically, sampling of reptiles, amphibians, aquatic animals, and birds remains severely inadequate relative to their diversity and ecological connectivity. Geographically, records from Yunnan and Tibet dominate, while western Sichuan, most of Guizhou, and the karst landscapes of Guangxi have been only sporadically surveyed. Addressing these taxonomic and geographic gaps is a priority for comprehensively understanding the region’s microbial dark matter and its implications for public health.

## 3 Novel microbial species in the environment

The natural environment serves as the primary reservoir for microbial dark matter, and its diversity is closely linked to environmental heterogeneity. Chen et al. (2022) conducted a metatranscriptomic analysis of 32 environmental samples from 16 provinces in China and identified 6,624 putative novel RNA viral taxonomic units belonging to 62 viral families, with sediments and animal feces emerging as environmental sources particularly rich in viral diversity.

Southwest China boasts diverse ecosystems ranging from high-altitude glaciers to geothermal hot springs, and from plateau lakes to forest soils, providing a rich array of habitats for the survival and evolution of microorganisms. A research team isolated more than 2,000 yeast strains from 1,200 samples of plants, soil, and seawater collected in Tibet and Yunnan, identified 462 species, and proposed 70 new yeast species within the Basidiomycota. They established 1 new family, 7 new genera, and 70 novel species, significantly expanding our understanding of yeast resources in the highland regions of Southwest China (Jiang et al., 2024).

In soil environments, 13 phosphorus-solubilizing fungi were isolated and screened from the air and soil of Yunnan, four of which were novel species (Doilom et al., 2020); a novel pigmented and heavy metal biosorbent bacterium isolated from the rhizosphere soil of *Epilobium hirsutum* L in Guizhou (Hou et al., 2018). In hot spring ecosystems, 36 novel species of the Chloroflexi were discovered in hot springs in Tibet and Yunnan; their metabolic characteristics suggest they can grow using a variety of low-molecular-weight organic compounds (Xian et al., 2020); another study isolated a novel *Clostridium* with high-efficiency lignocellulose degradation capabilities from hot springs in the Yunnan-Tibet region, providing a potential candidate strain for biofuel development (Liu et al., 2020).

In terms of microbial diversity in extreme environments, high-altitude water bodies and glacial environments have yielded equally significant results. A virome study of Nam Co Lake identified 742 viral species, including 383 new genera and 84 new families, revealing the high diversity of viruses in extreme environments (Wu et al., 2025); high diversity of the cold-adapted fungal genus *Cadophora* has been discovered in the glacial systems of the Qinghai-Tibet Plateau, and seven novel species have been identified (Zhang B. et al., 2022); metagenomic analysis of Bamucuo Lake reconstructed 75 near-complete genomes, 74 of which were derived from the water and represent entirely novel species (Wei et al., 2023). In short, the diverse natural habitats of Southwest China have given rise to a wealth of new microbial species, demonstrating the region’s immense potential as a treasure trove of microbial resources.

Beyond the discovery of novel microbial taxa, a global analysis of soil viromes has revealed that diverse vertebrate viruses are widely distributed across terrestrial ecosystems, and that viral diversity and relative abundance are markedly higher in agricultural soils than in natural habitats (Zhao et al., 2025). This finding is directly relevant to Southwest China, where the rapid expansion of cropland and plantation agriculture into formerly forested or grassland areas may increase the probability of human contact with soil-borne vertebrate viruses. Beyond pathogen-related risks, soil viral communities also play a critical functional role in ecosystem processes: a recent metagenomic analysis demonstrated that lytic viruses dominate soil viral communities and significantly influence soil organic carbon dynamics and ecosystem multifunctionality through modulation of microbial carbon use efficiency (Li S. et al., 2025). This dual significance–as both a reservoir of vertebrate-infecting viruses and a regulator of carbon cycling–underscores the importance of incorporating soil and environmental monitoring into regional microbial surveillance frameworks.

## 4 Emerging human pathogens

Based on the search strategy outlined in Table 1, we searched the PubMed, Web of Science, CNKI, and Wanfang databases for literature on emerging human pathogens in Southwest China since 2000, yielding 665, 560, 453, and 3,433 articles, respectively. After deduplicating the results using the Rayyan platform^1^, a total of 4141 articles were retained. The inclusion criteria were: (1) publication dates ranging from 2000 to March 26, 2026; (2) research content directly related to the search topic, covering aspects such as criteria for novel species identification and diagnostic techniques. Exclusion criteria included: (1) non-research literature (e.g., news reports, conference abstracts, reviews, etc.); (2) Studies not involving Southwest China, non-novel pathogens, or pathogens not causing human infections; (3) For multiple studies on the same novel pathogen, only the most comprehensive one was included.

**TABLE 1:** Strategies for identifying emerging human pathogens in Southwest China.

Database	Search terms
Web of Science	Topic = (((“southwest china” OR “yunnan” OR “guizhou” OR “sichuan” OR “xizang” OR “tibet”) AND (“emerging” OR “novel” OR “new” OR “first report” OR “new species” OR “previously unknown”) AND (“human” OR “patient” OR “case report” OR “outbreak” OR “infection” OR “seroprevalence” OR “zoonotic”) AND (“bacteria” OR “virus” OR “parasite” OR “fungi” OR “pathogen”))) AND Year Published = (2000–2026)
PubMed	((“southwest china”[Title/Abstract] OR “yunnan”[Title/Abstract] OR “guizhou”[Title/Abstract] OR “sichuan”[Title/Abstract] OR “tibet”[Title/Abstract] OR “xizang”[Title/Abstract])) AND ((“emerging”[Title/Abstract] OR “novel”[Title/Abstract] OR “new”[Title/Abstract] OR “previously unknown”[Title/Abstract] OR “first report”[Title/Abstract] OR “new species”[Title/Abstract])) AND ((“human”[Title/Abstract] OR “patient”[Title/Abstract] OR “case report”[Title/Abstract] OR “outbreak”[Title/Abstract] OR “infection”[Title/Abstract] OR “seroprevalence”[Title/Abstract] OR “antibody”[Title/Abstract] OR “zoonotic”[Title/Abstract])) AND ((“bacteria”[MeSH Terms] OR “viruses”[MeSH Terms] OR “parasites”[MeSH Terms] OR “fungi”[MeSH Terms] OR “bacterium”[Title/Abstract] OR “virus”[Title/Abstract] OR “parasite”[Title/Abstract] OR “fungus”[Title/Abstract])) AND (2000:2026[dp])
CNKI	Topic = Southwest China + Yunnan + Guizhou + Sichuan + Tibet AND bacteria + viruses + parasites + fungi + pathogens AND humans + patients + case reports + outbreaks + infections + seroprevalence + zoonotic AND emerging + novel + new + first reported cases + novel species + previously unknown
Wanfang	Topic = Southwest China + Yunnan + Guizhou + Sichuan + Tibet AND bacteria + viruses + parasites + fungi + pathogens AND humans + patients + case reports + outbreaks + infections + seroprevalence + zoonotic AND emerging + novel + new + first reported cases + novel species + previously unknown

After a thorough review of each article, four studies were ultimately included, reporting a total of four novel viruses with serological evidence of human infection: First, a novel rhabdovirus identified from Intermediate Horseshoe Bats in Yunnan Province, provisionally named Rhinolophus rhabdovirus DPuer (DPRV). Among 421 human serum samples with a history of fever collected near bat sampling sites, 20 (4.75%) tested positive by indirect immunofluorescence assay; of which 10 (2.38%) were confirmed to contain DPRV-specific neutralizing antibodies via the plaque reduction neutralization assay, suggesting the potential for bat-to-human transmission (Li et al., 2021). Second, the Plateau pika virus (PPV)–a mammalian virus first identified in Plateau Pikas on the Qinghai-Tibet Plateau–was detected in 8 (2.4%) of 335 serum samples collected from outpatients in Yushu, Qinghai, which tested positive for anti-PPV IgG antibodies (Luo et al., 2023). Similarly, two Chinese studies reported serological evidence of human infection with two other novel viruses discovered in Yunnan. One study documented the isolation of a variant of Banna virus (BAV) from 112 mosquito specimens in Yunnan; an IgG survey of 1,622 local residents revealed a seroprevalence rate of 3.8% (61/1,622) for BAV (Honghong, 2020). Another paper reported a novel hantavirus–Luxi hantavirus (LUXV)–isolated from *Eothenomys miletus* in Yunnan. In IgG surveys of small mammals and patients with fever of unknown origin in Yunnan, the seroprevalence rates were 18.46% (12/65) and 12.15% (13/107), respectively (Yunzhi, 2010). These findings collectively enrich the known spectrum of emerging zoonotic pathogens in southwestern China and confirm that humans have been exposed to these pathogens.

In addition to the aforementioned seropositive evidence, the search also identified four new pathogens directly isolated and identified from human samples. In the border region between Yunnan and Myanmar, a novel circovirus (Human-associated circovirus type 2, HuCV2) was identified in the blood of an intravenous drug user; this virus is closely related to porcine circovirus 3, suggesting the possibility of cross-species transmission via an unknown route; In a subsequent screening of 568 blood samples from intravenous drug users, HuCV2 sequences were detected again in another patient co-infected with HCV (Li Y. et al., 2023). In 2012, a novel species of the genus *Colpodella*, a parasitic protozoan, was discovered in the red blood cells of a female patient in Yunnan who had been suffering from a persistent cough (Yuan et al., 2012). In addition, regarding cultivable microorganisms, a novel species of *Nocardia*–*Nocardia huaxiensis* sp. nov. – was isolated from a skin biopsy sample taken from an elderly male patient at West China Hospital in Chengdu, Sichuan Province. The patient presented with erythematous nodules, pustules, and crusting on the dorsum of his left foot; the condition had persisted for 5 months (Zhuang et al., 2021). Interestingly, Xiang et al. (2025) conducted a retrospective study of 1,721 participants in Yunnan Province who presented with unexplained fever or anemia, and identified a novel *Babesia* species in three farmers exhibiting symptoms of infection, such as fever and anemia. In a follow-up study, researchers conducted a traceability investigation of livestock, wildlife, and tick specimens collected from the surrounding area, detecting pathogen sequences highly homologous to the human infection strain (Xiang et al., 2025).

In summary, through systematic literature searches and screening, this study identified a total of eight emerging pathogens with evidence of human infection in Southwest China (Table 2), covering three major taxonomic groups: viruses, bacteria, and parasites. Among these, four novel species of animal origin indicated a risk of human exposure based on serological surveys, while four were directly isolated from clinical samples. For some pathogens (such as the novel *Babesia* species), a complete chain of transmission has been established among hosts, vectors, and humans. These findings indicate that the southwestern region is a significant hotspot for emerging pathogens, necessitating the urgent and sustained strengthening of active surveillance and traceability research.

**TABLE 2:** Novel pathogens causing human infections in Southwest China.

Pathogen name	Pathogen type	Source	Pattern of origin	Evidence of human exposure/infection or detection	References
Rhinolophus rhabdovirus DPuer (DPRV)	Virus (Rhabdovirus)	Bat	Yunnan	Seropositivity rate: 4.75% (20/421), neutralizing antibody positivity rate: 2.38% (10/421)	Li et al., 2021
Plateau pika virus (PPV)	Virus (Arenaviridae)	Plateau pika	Tibet Plateau	Seropositivity rate: 2.4% (8/335)	Luo et al., 2023
Banna virus (BAV)	Virus (Sedoreoviridae)	Mosquitoes	Yunnan	The IgG antibody positivity rate was 3.8% (61/1622) using immunofluorescence	Honghong, 2020
Luxi hantavirus (LUXV)	Virus (Hantaviridae)	*Eothenomys miletus*	Yunnan	The positivity rate among patients with fever was 12.15% (13/107) using ELISA	Yunzhi, 2010
Human-associated circovirus type 2 (HuCV2)	Virus (Circoviridae)	Human blood	Yunnan-Myanmar Border	First detected in one intravenous drug user; subsequently, detected in one additional case among 568 samples by using quantitative PCR (1/568)	Li Y. et al., 2023
*Colpodella* spp. (untitled)	Parasite (*Colpodella*)	Human red blood cells	Yunnan	Detected in the red blood cells of a female patient with a chronic cough by using immunofluorescence	Yuan et al., 2012
*Nocardia huaxiensis* sp. nov.	Bacteria (Nocardiaceae)	Human skin tissue	Chengdu, Sichuan	One elderly male patient with a skin infection on the top of the foot	Zhuang et al., 2021
*Babesia* spp. (untitled)	Parasite (*Babesia*)	Human blood	Yunnan	Three cases were detected among 1,721 patients with fever or anemia (3/1,721) by using PCR	Xiang et al., 2025

## 5 Conclusion

As a global biodiversity hotspot, the complex and diverse ecosystems of Southwest China harbor an extraordinarily rich reservoir of microbial dark matter. Although this paper presents only a selection of research findings, it clearly reveals the astonishing diversity, unique distribution patterns, and potential public health risks associated with microbial dark matter in this region, underscoring its significant research value and strategic importance.

**FIGURE 1:**
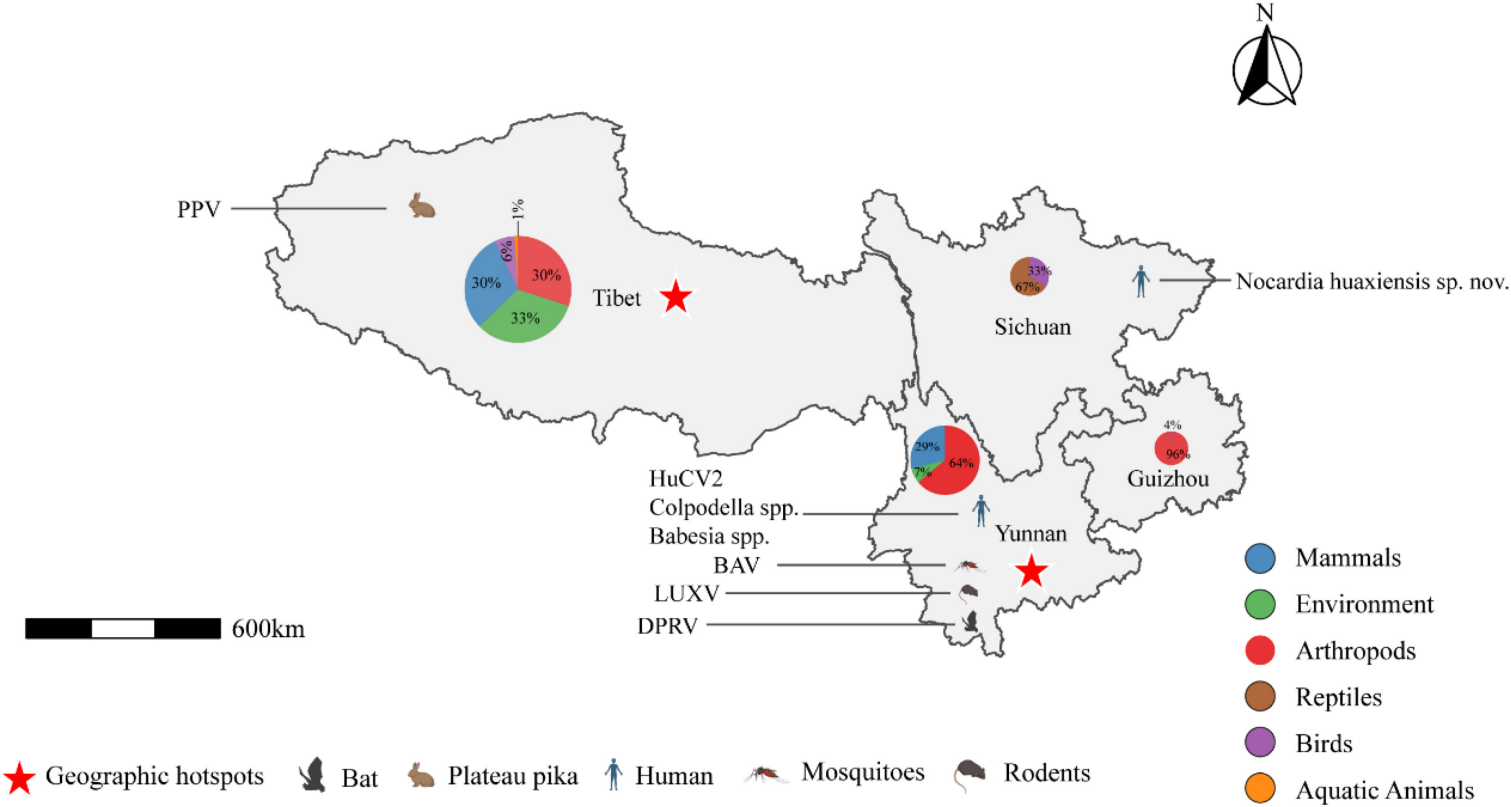
Map of novel species distribution in Southwest China. This map is drawn based on the standard map with drawing review number GS (2024) No. 0650. The figure highlights geographic hotspots (red stars) for the discovery of novel microbial species in southwest China, major host groups (pie chart), and novel human pathogens. The data are sourced from the literature cited in this review, and the figure provides a visual representation of the southwest region as a hotspot for emerging infectious diseases.

From a geographical perspective (Figure 1), Yunnan Province is a key hotspot for novel species discoveries in Southwest China; this study documented approximately 700 novel species, encompassing arthropods, mammals, and environmental samples. The diversity of viruses carried by vectors such as mosquitoes, ticks, and midges was particularly striking, while bats–as important reservoirs of pathogens such as coronaviruses–also contributed to several significant discoveries. The Tibet Autonomous Region, with its extremely cold and high-altitude environment, leads in the number of novel species. Environmental samples–such as viruses from Lake Nam Co and fungi from glaciers–as well as mammals and arthropods all exhibit extremely high diversity, highlighting the Qinghai-Tibet Plateau’s status as a unique repository of microbial resources. Guizhou Province has a relatively small number of novel species, primarily from arthropods. Sporadic novel species have also been discovered in Sichuan Province and the Guangxi Zhuang Autonomous Region, though the depth and breadth of research in these areas do not yet match those of the Yunnan-Tibet region.

When categorized by host type (Figure 1), novel species associated with arthropods were the most abundant, encompassing mosquitoes, ticks, midges, fleas, and mites, indicating the central role of vectors in the reservoir and transmission of pathogens. Novel species associated with mammals ranked second, with the viral diversity carried by hosts such as bats, rodents, and plateau pikas being particularly notable; among these, there were groups closely associated with human diseases (such as SARS-related coronaviruses, henipaviruses, and hantavirus). Novel species associated with birds, aquatic animals, and reptiles were relatively fewer in number, but their potential as reservoirs for microorganisms has begun to emerge. Novel species in environmental samples also exceeded 900; new bacterial, fungal, and viral species were widely found in environments ranging from hot springs, glaciers, and high-altitude lakes to forest soils, revealing the adaptive evolutionary potential of microorganisms in extreme environments.

Since the beginning of the 21st century, the international community has faced several major outbreaks of emerging infectious diseases, including the 2009 H1N1 pandemic, the 2014 Ebola outbreak in West Africa, the 2020 COVID-19 pandemic, and the 2022 monkeypox outbreak, demonstrating that the threat of emerging infectious diseases remains ever-present. The World Health Organization has also updated its “List of Pathogens of Priority Concern” on multiple occasions, adding more than 30 pathogens–including monkeypox virus, influenza A virus, and dengue virus–that could trigger future public health crises (Mallapaty, 2024). In light of this situation, we must remain highly vigilant against unknown pathogens, with a particular focus on those that have previously infected humans.

The complex ecosystems of southwest China harbor a wide variety of potential pathogens. Although the reports of direct human infections summarized in this paper are limited, their potential pathogenicity should not be overlooked. In terms of pathogen distribution patterns, blood-feeding arthropods and various mammals constitute the core reservoirs of pathogens. Among these, bats and rodents, due to their high species diversity and significant overlap with human activity areas, have become the primary natural hosts for emerging pathogens. In terms of spatial distribution, ecologically transitional zones such as Yunnan Province, the Qinghai-Tibet Plateau, and the China-Myanmar border are the regions with the highest concentration of new pathogen discoveries, forming natural “hotspots” for pathogen evolution and spillover. In terms of pathogen types, RNA viruses dominate, encompassing high-priority groups such as coronaviruses, henipaviruses, and arenaviridae. Meanwhile, bacterial pathogens such as rickettsiae and ehrlichiae, as well as eukaryotic pathogens such as *Babesia* and *Echinococcus*, are also frequently identified.

The risk of cross-species transmission is a prominent feature of potential pathogens in this region. Multiple serological surveys and functional experiments have confirmed that certain emerging viruses (such as the bat-derived DPRV and the plateau pika-derived PPV) possess the ability to infect human or mammalian cells. Furthermore, the detection of specific antibodies in local populations has elevated these pathogens from a “potential risk” to a “real threat.” It is worth noting that livestock farming and animal trade significantly increase the risk of pathogen transmission; most emerging infectious diseases originate from animals, and the wildlife trade greatly amplifies this risk (Shivaprakash et al., 2021). For example, 125 viruses were identified in 461 farmed fur animals that died from disease, including 36 novel viruses and 39 high-risk viruses with the potential for cross-species transmission (Zhao et al., 2024); among the 1,941 wild animals traded, 102 viruses that infect mammals were identified, including 21 novel species considered to pose a high potential risk to humans and livestock (He et al., 2022). Taken together, these findings underscore the need to strengthen surveillance of pathogens in non-traditional livestock and wildlife.

The rich microbial diversity of the Southwest region not only harbors a vast number of unknown pathogens, but also provides a vital protective barrier for regional health through complex ecological mechanisms. Highly diverse microbial communities constitute a defense system in and of themselves. According to the principle of competitive exclusion, a saturated and stable microbial ecosystem can effectively occupy various ecological niches and consume available resources, thereby forming a strong resistance to the colonization of newly invading pathogens (Leung and Weitz, 2019). Furthermore, the native microbial communities of *Anopheles* mosquitoes can prevent the establishment of introduced *Wolbachia* infections, and highly diverse indigenous microbial communities can also serve as natural barriers against pathogen invasion (Hughes et al., 2014). These findings are consistent with the “dilution effect hypothesis,” which posits that higher microbial biodiversity can reduce the risk of transmission per capita by limiting the probability of successful pathogen colonization (Keesing et al., 2006). Therefore, exploration of the microbial dark matter in the Southwest should not focus solely on its role as a “threat,” but should also delve deeply into its ecological function as a “guardian.” This is crucial for formulating comprehensive risk prevention and control strategies that balance ecological conservation and public health.

Overall, these findings have not only significantly expanded our understanding of microbial diversity in China, but also revealed the potential risks associated with the southwestern region as a “hotspot” for emerging zoonotic diseases. The discovery of a large number of novel viruses in wild animals such as bats and rodents, as well as in vector arthropods, coupled with increased cross-border ecological connectivity and human activities, means that the risk of cross-species transmission and spillover of pathogens in this region cannot be ignored. Therefore, future research should prioritize systematic, long-term monitoring at the ecological interfaces identified in this study, combining environmental sampling of soil, water, and vectors with serological surveys of sentinel animals and humans, and strengthening cross-border data sharing to track the spread of pathogens across ecologically connected landscapes. These measures are essential for translating the region’s documented microbial diversity into actionable early-warning capabilities.

## Footnotes

1
https://new.rayyan.ai/
